# Respiratory Manifestations of Strongyloidiasis: Significance of Diagnosis, Treatment, and Screening

**DOI:** 10.7759/cureus.80557

**Published:** 2025-03-14

**Authors:** Maja Banjac, Ivana Vujovic, Aleksandra Colovic Popadic, Milica Devrnja, Dusanka S Obradovic

**Affiliations:** 1 Critical Care Department, Internal Medicine Department, Institute for Pulmonary Diseases of Vojvodina, Sremska Kamenica, SRB; 2 High Dependency Unit, Internal Medicine Department, Institute for Pulmonary Diseases of Vojvodina, Sremska Kamenica, SRB; 3 Center for Microbiology, Institute of Public Health of Vojvodina, Novi Sad, SRB; 4 Faculty of Medicine Department, Universitiy of Novi Sad, Novi Sad, SRB

**Keywords:** diarrhea, hyperinfection syndrome, pneumonia, screening, strongyloides stercoralis

## Abstract

Strongyloidiasis is a widespread disease characterized clinically by acute or chronic manifestations, which, in high-risk individuals and immunocompromised individuals, can progress to hyperinfection syndrome and disseminated forms with significant mortality rates. Among extraintestinal organs, the lungs are most commonly affected. Clinical presentations are very diverse, contributing to the challenges of diagnosis and timely treatment initiation. We present the case of a 57-year-old patient who was admitted to the intensive care unit (ICU) due to bilateral pneumonia and septic shock. During a two-month hospitalization period, the patient spent 16 days on invasive mechanical ventilation. Initially, nonspecific symptoms, such as weakness, fatigue, and cough, evolved into complications, including diarrheal syndrome, hemoptysis, urticaria, and ventilator-associated pneumonia (VAP) caused by Gram-negative bacteria. Numerous larvae of *Strongyloides stercoralis* were identified in a stool sample. In addition to antibiotic therapy, antiparasitic treatment was administered, resulting in significant improvement in the patient's overall condition, leading to discharge for further home care. In this case, community-acquired pneumonia was complicated by the reactivation of chronic strongyloidiasis in the form of hyperinfection syndrome in a high-risk patient. High-risk individuals and immunocompromised individuals should undergo screening for parasitic infections in cases presenting with gastrointestinal symptoms, particularly diarrhea and respiratory symptoms. Screening for parasitic infections should be integrated into routine clinical practice for this population.

## Introduction

Strongyloidiasis is a widely prevalent intestinal nematode infection caused by *Strongyloides stercoralis (S. stercoralis)*. It has been estimated that 600 million people worldwide suffer from this condition annually and, due to migration and tourism, the disease is becoming more widespread [1].

Parasite's Life Cycle

The parasitic life cycle of this primarily human parasite begins with the entry of infective filariform larvae into the human body through intact, exposed skin from contaminated water or soil. They circulate to the right heart and then into the lungs, inducing cough, after which they are swallowed and reach the digestive system. In the small intestine, the larva matures into an adult female that lays eggs, giving rise to rhabditiform larvae, which can further cause autoinfection or be excreted in the stool [2].

Clinical Manifestations

Strongyloidiasis can manifest as acute, chronic, hyperinfection, or disseminated form, and in most cases, the disease is oligosymptomatic. At the site of entry, parasite can cause dermatitis known as "larva currens" [1]. Symptoms similar to Loeffler's syndrome may occur due to the parasite's migration through the lungs, while symptoms affecting the upper gastrointestinal tract can include fever, diarrhea, abdominal pain, and urticaria. However, conditions such as human immunodeficiency (HIV) and human T-lymphotropic virus (HTLV), prolonged corticosteroid therapy, malnutrition, alcoholism, and other immunocompromised conditions can lead to uncontrolled parasite multiplication and life-threatening dissemination of the parasite as part of hyperinfection syndrome and disseminated forms, with the mortality rate of 85% [3,4]. The lungs are the most commonly affected extraintestinal organ in strongyloidiasis [5]. In addition to the previously mentioned respiratory symptoms, hyperinfection can lead to the development of respiratory failure and acute respiratory distress syndrome (ARDS), which is often complicated by severe secondary bacterial and fungal infections [6]. Secondary bacterial infections are almost always caused by Gram-negative bacteria from the *Enterobacterales* family [7]. Hemoptysis occurs in 10% of patients. Diffuse alveolar hemorrhage, which often has a fatal outcome, is also possible [8]. The radiological manifestation of the disease is nonspecific and ranges from normal lung parenchyma findings to patchy opacities, lobar pneumonia, or diffuse interstitial changes described on computed tomography (CT) as ground glass opacities (GGO) [3,6]. Eosinophilia, commonly found in chronic strongyloidiasis, may be absent in cases of hyperinfection and disseminated disease due to corticosteroid therapy, cytostatics, or secondary bacterial infections [4].

Microbiological Confirmation of Disease

Diagnosing strongyloidiasis is challenging as the infection is often asymptomatic or accompanied by nonspecific gastrointestinal symptoms. Microscopic examination of at least three stool samples is the most frequently used method for diagnosis. Serological methods cannot reliably distinguish current from past infections and are not applied in routine diagnostics [9].

## Case presentation

**Figure 1:**
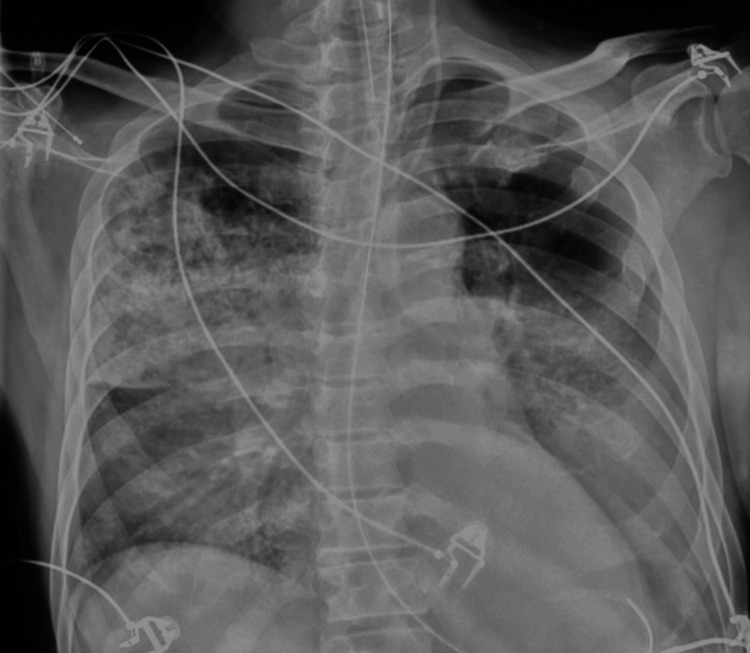
Chest X-ray on the day of admission indicates bilateral, predominantly right-sided strip-like patchy pneumonia infiltrates, as well as decreased transparency in the left basal region, where a small amount of pleural effusion was verified by ultrasound.

**Figure 2:**
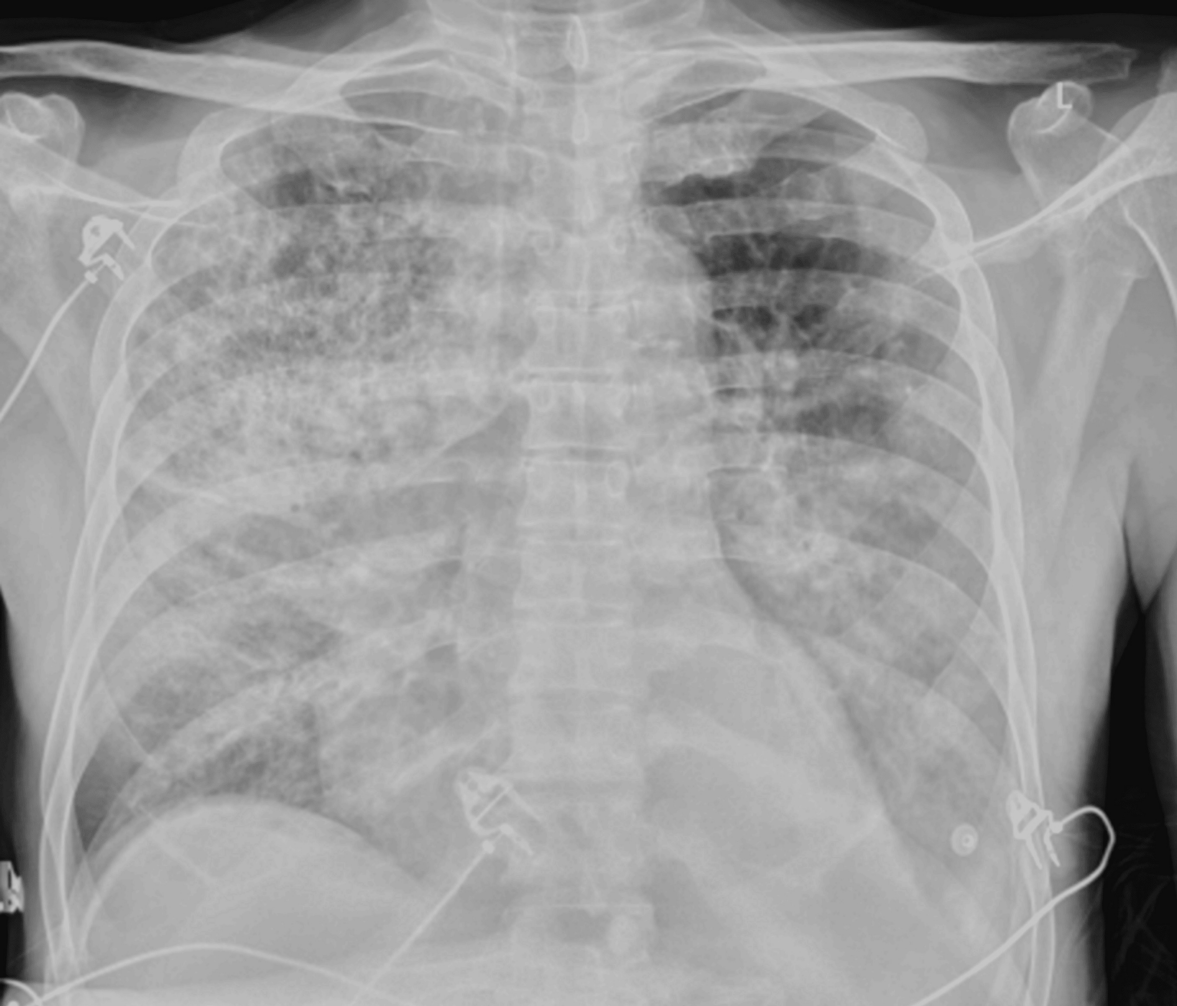
Chest X-ray on the sixth day of hospitalization indicates bilateral progression of fine mottled changes, which was accompanied by clinical deterioration and the need for reintubation.

A 57-year-old male patient was admitted to the intensive care unit at the Institute for Pulmonary Diseases of Vojvodina due to severe hypoxemic respiratory failure (P/F 140) and septic shock secondary to bilateral pneumonia, which required the use of invasive mechanical ventilation, vasoactive support, and continuous renal replacement therapy. The illness began with symptoms of weakness, fatigue, and fever. Subsequently, it was reported that the patient had occasional symptoms of cough and watery stools. The patient had a history of hypertension and additionally reported consuming large quantities of alcohol daily, specifically 24 standard drinks per day. He denied the use of immunosuppressive drugs and HIV antibodies were negative upon testing. He worked in a bridge construction company, and he lived in collective accommodation. Upon admission, a differential blood count revealed eosinopenia alongside a normal absolute white blood cell count, as well as significantly elevated markers of inflammation (Table 1). Chest X-rays showed diffuse streaky-patchy pneumonic infiltrations (Figures 1-2).

**Table 1:** Inflammatory marker values in the patient's serum upon admission to the intensive care unit. CRP: C-reactive protein; PCT: procalcitonin

Items	The patient's serum values	Reference values
Leukocytes	9.5 x 10^9^/L	4.0-11.0 x 10^9^/L
Neutrophils	9.11 x 10^9^/L	2.0-7.6 x 10^9^/L
Lymphocytes	0.21 x 10^9^/L	1.0-4.5 x 10^9^/L
Eosinophils	0.0 x 10^9^/L	0.1-0.6 x 10^9^/L
CRP	327.8 mg/L	<5.0 mg/L
PCT	42.31 ng/mL	<0.05 ng/mL

From microbiological analyses taken upon admission, blood cultures were positive for *Streptococcus pneumoniae*, while the tracheal aspirate showed no bacterial growth. On the fifth day of hospitalization, after the patient's condition had stabilized, he was extubated. However, a few days later, the patient's general condition deteriorated, with altered consciousness, the emergence of a diffuse maculopapular skin rash, hemoptysis, and respiratory failure worsening, requiring reintubation. Due to the necessity for prolonged mechanical ventilation, a percutaneous tracheostomy was performed during the hospitalization.

In the initial days of hospitalization, watery diarrhea appeared, which was tested multiple times for the presence of *Clostridioides difficile*, with negative results. The clinical picture worsened with the development of profuse, frequent, watery stools, significant weight loss, and biochemical signs of malnutrition. Simultaneously, in addition to elevated CRP values ​​and an increase in total leukocyte values, there was an increase in eosinophil levels, which reached 13 times the upper reference range value (8.54 x 10^9^/L, reference value 0.1-0.6 x 10^9^/L) (Table 2).

**Table 2:** Inflammatory marker values at the onset of worsening patient's condition and suspected parasitic infection. CRP: C-reactive protein

Items	The patient's serum values	Reference values
Leukocytes	19.0 x 10^9^/L	4.0-11.0 x 10^9^/L
Neutrophils	8.70 x 10^9^/L	2.0-7.6 x 10^9^/L
Lymphocytes	1.02 x 10^9^/L	1.0-4.5 x 10^9^/L
Eosinophils	8.54 x 10^9^/L	0.1-0.6 x 10^9^/L
CRP	185.0 mg/L	<5.0 mg/L

**Figure 3:**
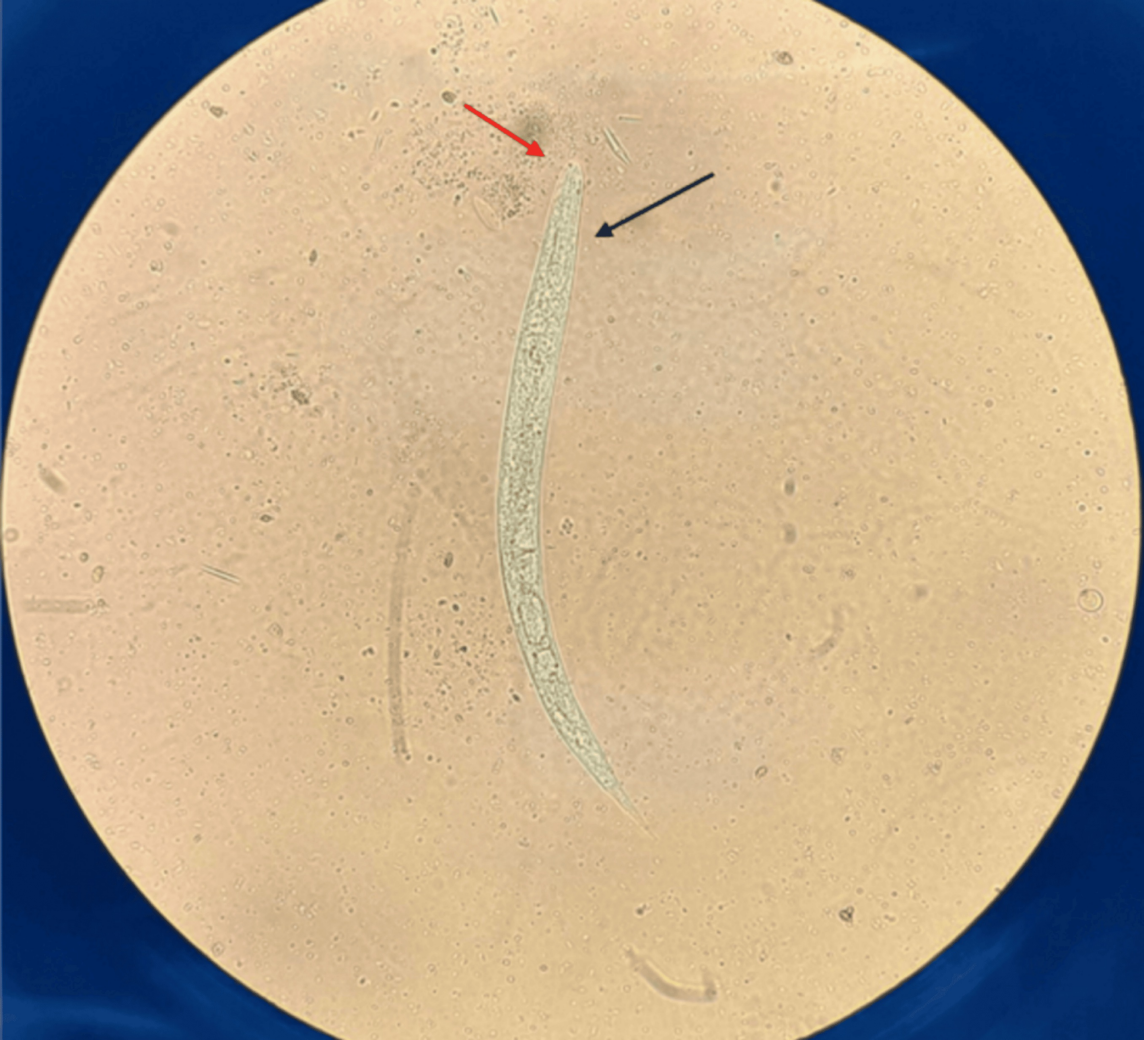
Larva of S. stercoralis in an unstained wet mount of stool sediment (400x). The red arrow indicates a short buccal canal, and the black arrow indicates a rhabditoid esophagus.

**Figure 4:**
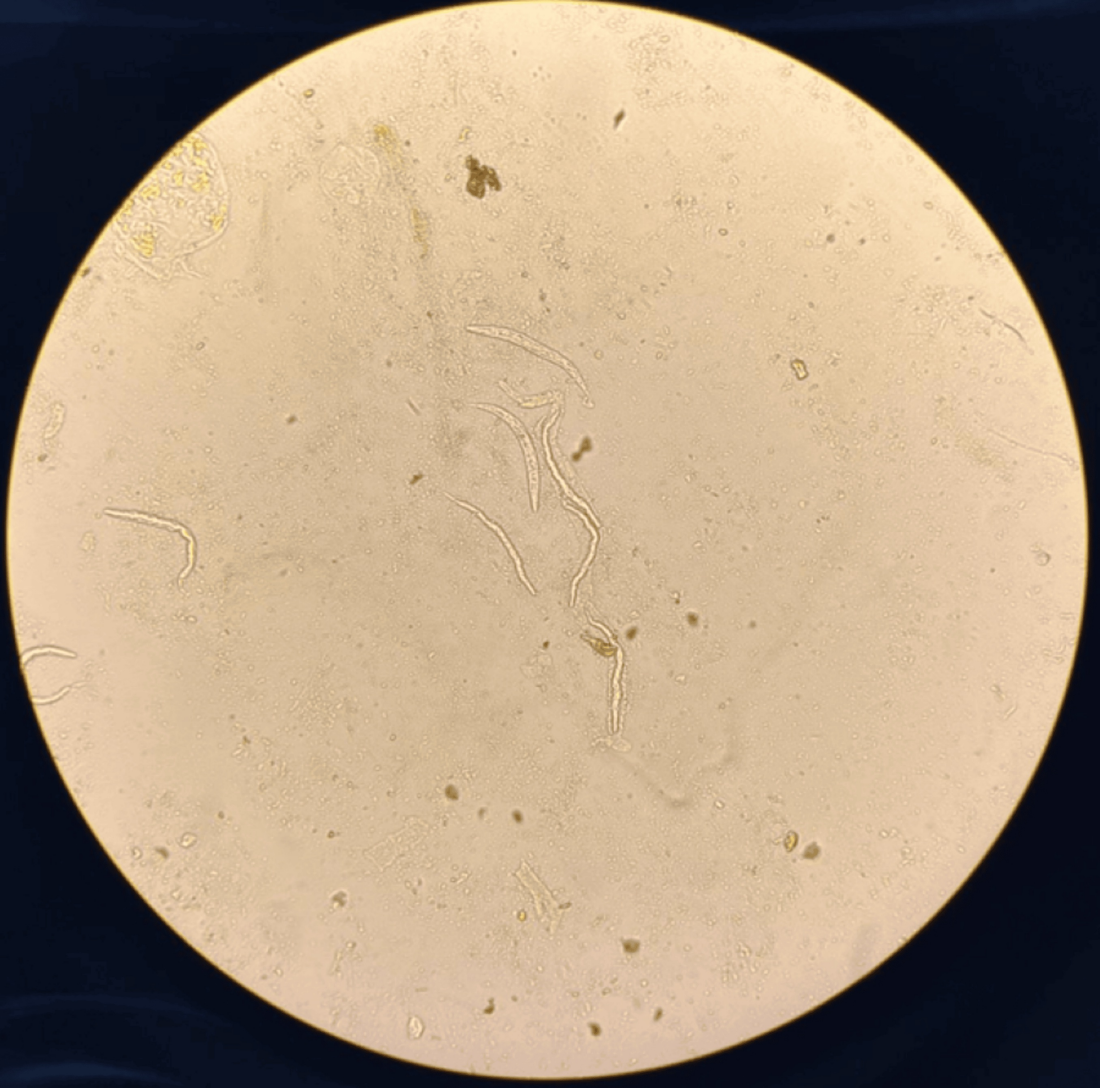
Presence of multiple S. stercoralis larvae in an unstained wet mount of stool sediment (100x).

At that point, stool samples were tested for the presence of parasites. Examination of the direct native microscopic slide revealed a large number of motile larvae. Based on their characteristic morphology, primarily characterized by a short buccal cavity and a clearly pointed tail, the presence of rhabditiform larvae of *S. stercoralis* was confirmed (Figures 3-4). Examination of several consecutive stool samples did not demonstrate the presence of filariform larvae of this parasite.

Antiparasitic therapy with mebendazole was initiated, due to inadequate pharmacy supplies of albendazole, without the complete resolution of symptoms. Therefore, ivermectin was added to the treatment regimen due to suspicion of hyperinfection syndrome, despite the absence of parasite larvae in tracheal aspirate samples. Nevertheless, this does not rule out the possibility of a hyperinfection syndrome as other clinical parameters can indicate such a condition.

**Figure 5:**
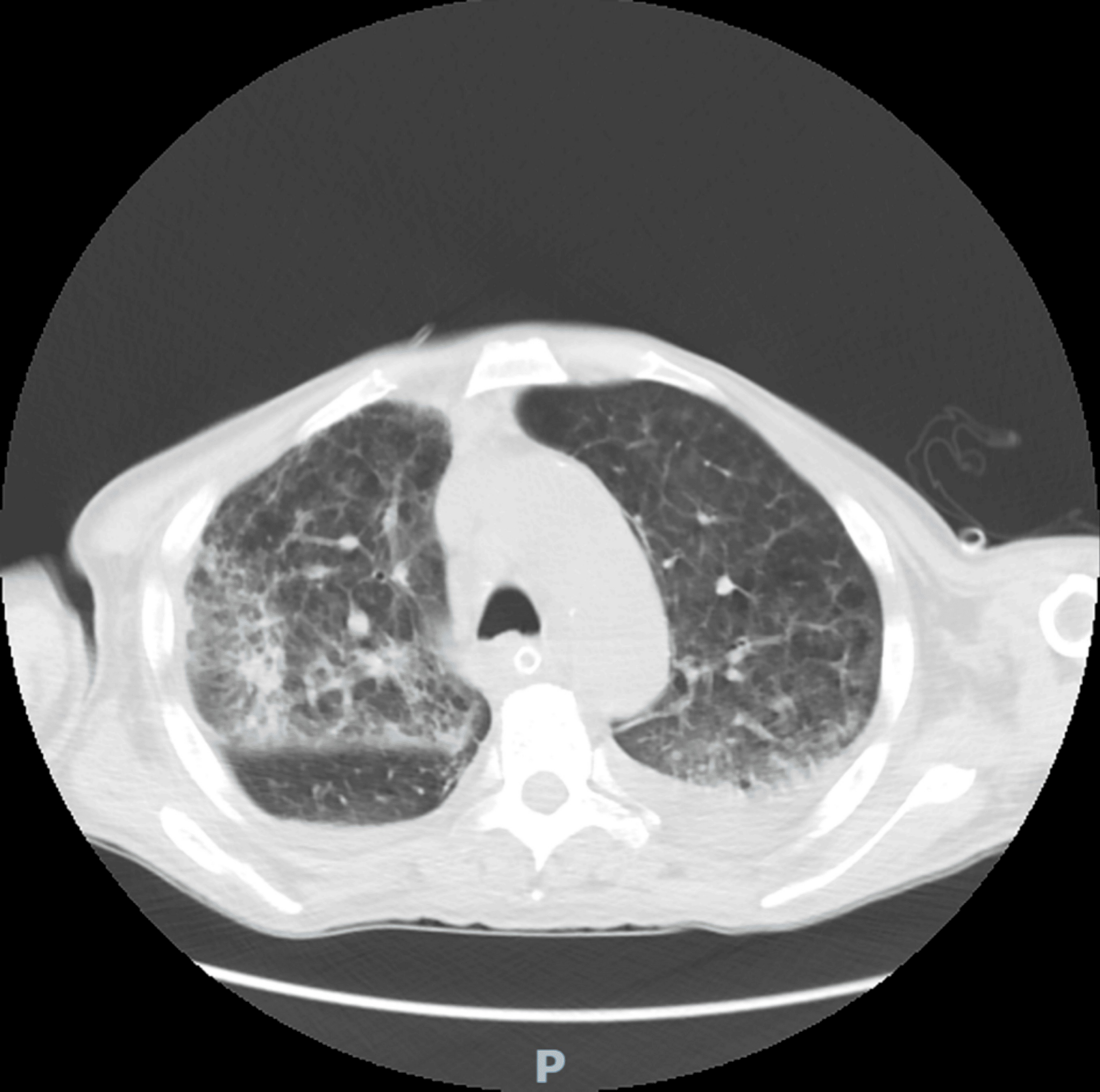
The chest CT scan performed one month after admission described bilateral irregular consolidations with an air bronchogram, primarily of inflammatory etiology, with signs of perilesional bronchiolitis, and then bilateral reticular changes in the interstitium and ground-glass opacity (GGO) zones.

Initially, the patient was treated with ceftriaxone, a third-generation cephalosporin, for community-acquired pneumonia. Later, due to the development of ventilator-associated pneumonia (VAP), antibiotics were adjusted according to the antibiogram for the isolated hospital-acquired bacteria (*Acinetobacter baumannii *complex*, Klebsiella pneumoniae*) and the latest Infectious Diseases Society of America (IDSA) guidance on the treatment of antimicrobial-resistant Gram-negative infections. These included high-dose ampicillin-sulbactam and polymyxin B for *A. baumannii *complex and imipenem-cilastatin for *K. pneumoniae*. The laboratory findings revealed significantly elevated levels of immunoglobulin E (IgE), which plays a crucial role in the immune response to parasite infections (IgE 643.06 kU/L, reference value <100 kU/L). A chest computed tomography (CT) scan was performed, revealing bilateral consolidations primarily of inflammatory origin with air bronchograms and perilesional signs of bronchiolitis. Additionally, bilateral reticular interstitial changes and GGOs were described (Figure 5).

Due to altered consciousness during hospitalization, a CT of the brain was also performed, which showed no acute pathological changes. After two months of hospitalization, the patient was discharged in improved general condition, with recommendations for additional therapy, personal hygiene, and dietary management. Spirometry performed at a follow-up examination revealed a moderate form of obstructive lung ventilation disorder. Following the completion of eradication therapy and confirmation of negative stool samples for parasites, therapy for chronic obstructive pulmonary disease was prescribed.

## Discussion

The patient presented in this case is a 57-year-old male individual, still of working age, who was treated in the ICU for an extended period due to bilateral pneumonia. In retrospect, we assume that the patient experienced an acute exacerbation of chronic strongyloidiasis in hyperinfection form. In our patient's case, the infection was confirmed through stool sample analysis. However, a limitation was the unconfirmed presence of parasites in the lungs as the tracheal aspirate tested negative for parasites. Nonetheless, the false-negative result could be explained by the late sampling during the initiation of antiparasitic therapy and potentially inadequate sampling methods.

Among the risk factors for strongyloidiasis, the patient primarily had a lifestyle that involved working in damp soil and daily alcohol consumption in larger quantities, as we mentioned, about 24 standard drinks a day. The occurrence of hyperinfection and disseminated forms of strongyloidiasis in individuals with alcohol use disorder is not uncommon. If alcoholism is associated with poor hygienic conditions, the risk of infection and subsequent autoinfection is increased, which contributes to the general harmful effect of alcohol on the body. It has been shown that ethanol, through vagal stimulation and damage to the morphology of intestinal microvilli, leads to decreased intestinal motility, allowing the rhabditiform larva to remain in the intestinal tract for a longer period, which increases the possibility of autoinfection [10,11]. The impact of alcohol abuse on the immune system is complex, involving both innate and acquired immune responses. It is known that patients with alcoholism have a reduced number of macrophages, impaired T lymphocyte function, and a decreased level of IgA antibodies, which can lead to ineffective defense against infectious agents in general [10-12]. A direct pathway through which alcoholism promotes severe strongyloidiasis is the modification of steroid metabolism, leading to elevated endogenous steroid levels in the body. Certain steroid metabolites resemble ecdysteroids, which act as growth factors for *S. stercoralis* and improve the fertility of female parasites, consequently increasing the number of rhabditiform larvae and enabling autoinfection [10,12].

Strongyloidiasis is not a rare condition but is often overlooked, especially due to the variability in clinical manifestations and diagnostic challenges. As the gastrointestinal tract is the dominant organ system in the parasite's life cycle, symptoms related to it are almost inevitable and should raise suspicion of a parasitic infection, particularly in individuals with risk factors [3,13]. In our case, diarrhea and eosinophilia were crucial in raising suspicion of a parasitic infection. Subsequently, confirmation of the infection was followed by other symptoms, such as diffuse urticaria, hemoptysis, and worsening respiratory problems, which fit the picture of hyperinfection. It is challenging to differentiate whether the worsening of pneumonia caused by Gram-negative bacteria is a consequence of intubation and ICU stay or if the primary cause is the migration of parasites from the intestines to the lungs due to the breakdown of the normal gut mucosal barrier [13]. Regardless of the primary cause, we believe that the exacerbation of strongyloidiasis contributed to the severity of the clinical presentation, the duration of mechanical ventilation, and the length of hospitalization.

Several case reports in the literature have described cases similar to ours. Nabeya et al. published a review article describing 16 cases of strongyloidiasis, of which 15 presented with respiratory manifestations and five of them died. Among these 15 cases, eight exhibited clinical features of ARDS and in seven cases secondary bacterial infections with Gram-negative bacteria (e.g., *A. baumannii, K. pneumoniae, Escherichia coli, and Citrobacter koseri*) were confirmed. Strip-like opacities within pneumonic infiltrations were the most common radiological finding, with ground-glass opacities (GGO) also frequently described on chest CT scans [7]. Another case report by Khadka et al. [14] involved a patient hospitalized for symptoms, including dyspnea, cough, hemoptysis, vomiting, nausea, and abdominal cramps. The patient had been receiving treatment for acute gastritis and exacerbation of chronic obstructive pulmonary disease. Corticosteroid therapy was initiated, leading to further deterioration and subsequent transfer to the ICU, where the chest X-ray revealed massive right-sided pneumonic infiltrations. Strongyloidiasis was confirmed through stool and bronchoalveolar lavage samples and antiparasitic therapy was initiated, but the patient passed away shortly afterward [14]. Dogan et al. presented the case of a 17-year-old girl with symptoms including weakness, fever, cough, and weight loss, with a preserved immune system and no comorbidities. A chest CT revealed diffuse micronodular changes and GGO. Infection with *S. stercoralis* was confirmed by pathological analysis of materials obtained through transbronchial biopsy, followed by direct examination of a stool sample. The patient presented herein had barefoot soil contact in a holiday resort and a habit of eating clams [15]. Bronchoscopy with material sampling, which could have increased the likelihood of confirming the presence of parasites in the lungs, was not performed in our case.

In Europe, there are no official guidelines or recommendations for screening for strongyloidiasis, except in Ireland and the United Kingdom, where general guidance for screening and treatment of infectious diseases in asymptomatic patients has been introduced due to the high number of migrants. Therefore, the prevalence of parasitosis in Europe cannot be precisely determined, and insights are primarily based on individual studies (less than 5% in Italy and Spain). The European Centre for Disease Prevention and Control (ECDC), in its *Guidance for Screening and Vaccination of Infectious Diseases in Newly Arrived Migrants in the EU/EEA*, recommends screening for antibodies to strongyloidiasis in non-endemic countries because of its higher sensitivity compared to conventional parasitological methods (among these methods, agar plate culture and Baermann methods are emphasized) [16]. Similarly to Europe, there are no precise data on the prevalence of this parasitosis in Serbia. A study conducted in Belgrade covered a 30-year period (1993-2023), with the aim of monitoring the presence of the parasite in soil samples from green areas in the city from April to September, where *S. stercoralis* happened to be only the seventh most common of the 11 detected parasite species [17]. However, a similar study from 2018 conducted in the Kruševac area shows that the prevalence of this parasite in urban green areas in Kruševac is significant in spring, under moist and warm climatic conditions [18].

Although pneumonia is most often caused by bacteria, viruses, and sometimes fungi, we should not forget about parasites as a possible etiological factor. Considering all of the above, it can be concluded that strongyloidiasis is a challenging condition to diagnose. Timely and appropriate therapy significantly reduces complications and mortality associated with severe forms of the disease. Our goal is to raise awareness about the necessity of screening for strongyloidiasis and other parasitic infections in immunocompromised patients, whether as a part of their underlying condition or secondary to corticosteroid or other immunosuppressive therapies. Screening should be implemented as a routine clinical practice in immunocompromised patients to reduce the complications of the disease and decrease mortality rates, as well as to shorten hospital stays [19,20].

## Conclusions

Respiratory symptoms joined with gastrointestinal symptomatology in individuals with poor living conditions, alcohol or drug abuse, prolonged corticosteroid therapy, and other immunocompromised conditions should be investigated for the presence of parasitic infections, including *S. stercoralis*.

A diarrheal syndrome of unclear etiology, especially in the early days of hospitalization without the influence of prolonged antibiotic therapy and despite the absence of eosinophilia, should be investigated for parasitic infections. Incorporating screening for parasitic infections into standard clinical practice for immunocompromised patients is imperative. This proactive measure aims to mitigate disease complications, decrease mortality rates, and shorten hospital stays.
